# Immunotoxic effect of thiamethoxam in immunized mice with *Brucella abortus* cultural filtrate antigen

**DOI:** 10.14202/vetworld.2016.1407-1412

**Published:** 2016-12-13

**Authors:** L. H. Salema, M. J. Alwan, Afaf Abdulrahman Yousif

**Affiliations:** 1 Department of Pathology and Poultry Diseases, College of Veterinary Medicine, University of Baghdad, Iraq; 2 Ministry of High Education and Scientific Research, Baghdad, Iraq; 3 Department of Internal and Preventive Veterinary Medicine, University of Baghdad, Baghdad, Iraq

**Keywords:** *Brucella abortus*, enzyme-linked immunosorbent assay test, interferon gamma, phagocyte assay, skin test, thiamethoxam

## Abstract

**Aim:**

This study was planned for determination the toxic effect of thiamethoxam (TMX) in immunized mice with *Brucella abortus* culture filtrate antigen (CFBAgs) (as a vaccine) and its role of TMX on decrease activity of *B. abortus* antigen on eliciting of humoral and cellular immunity.

**Materials and Methods:**

To achieve these goals 60 female mice were used, 7-8 weeks age, they were divided equally into three groups (20 in each group) and treated as follows: 1^st^ group: Mice were immunized with CFBAgs intraperitoneally in two doses, 2 weeks intervals with (protein concentration 2 mg\ml), 2^nd^ group: Mice immunized as in the 1^st^ group and was administrated orally with 1/10 lethal dose 50% of TMX (83.7 mg/kg B.W.) for 4 weeks daily, 3^rd^ group was administrated orally with 0.3 ml normal saline served as a control group. At day 28 post immunization (PI) delayed type hypersensitivity (skin test) was done, and serum samples were collected at day 30 (PI) for detection of passive hemagglutination test (PHA); interferon gamma (IFN-γ) which was done by enzyme-linked immunosorbent assay test in addition to phagocytes assay.

**Results:**

The results of skin test post injection with soluble antigen of *B. abortus* intradermally showed a high significantly mean values at p≤0.05 of footpad skin thickness in the 1^st^ group of mice which recorded (0.51±0.002 mm) as compared with the 2^nd^ group of mice which showed (0.08±0.002 mm) after 24 h; the mean values of skin thickness were declined in the 1^st^ mice (0.46±0.002) and 2^nd^ mice (0.070±0.001) at 48 h; control group showed a negative results. These results were agreed with results of serum levels of IFN-γ (pg/ml) that showed that a significant increase the vaccinated 1^st^ group (406.36±1.52), than those values in the 2^nd^ group (151.61±0.89) and negative result in 3^rd^ group (46.47±0.60), in addition to results of PHA test which showed a significant increase in antibody titer in the 1^st^ group (139±12.16) with low level of serum antibody in the 2^nd^ group (7.66±0.33). Phagocytic ratio results in the 1^st^ group showed an increase to reach (18.55±0.44) than a ratio in the 2^nd^ group (13.24±0.32) and the control group (5.46±0.25).

**Conclusion:**

It was concluded that TMX induced suppression of humoral and cellular immune responses in immunized mice with CFBAgs.

## Introduction

Neonicotinoid currently dominates the insecticides market as a seed treatment on Canada’s major prairie crop. Thiamethoxam (TMX) followed clothianidin where the dominant seed treatment by mass and area [1]. Ford and Casida [2] investigated that neonicotinoid pesticides from 17% of all global market insecticides due to their physiological properties such as less resident in the environment and animal tissues as well as low toxicity to mammals. Kurwadkar *et al*. [3] reported that environmental presence and retention of commonly used neonicotinoid insecticides such as dinotefuran, imidacloprid (IMD), and thiamethoxam (TMX) are a cause for concern and prevention because to their potential toxicity to non-target species.

The low affinity of neonicotinoids such as TMX for vertebrate relative to insect nicotinic receptors is the major factor in their favorable toxicological profile [4]. Most of the studies on pesticide toxicity have been focused on enzyme alteration, mutagenic, carcinogenic potential of these agents and gross pathological changes, the effects of pesticides on the immune response have received attention now, it is now clear that changes in host immunity may occur after pesticide ingestion. Furthermore, a few study on effects of TMX (second generation neonicotinoid) on the immune system but they find immunotoxic alterations of lead acetate after exposure with TMX in animal model [5].

Pesticides, such as neonicotinoids, can exert inhibitory action on nuclear factor kappa B activation and its expression is down-regulated to allow an inducible immune response which was responsible for the production of pro-inflammatory cytokines [6], therefore, pesticides induced altered innate immune response which was essential in stimulated cell-mediated immune response and in control of intracellular pathogens such as *Brucella* infection.

*Brucella abortus* vaccines play a role in bovine brucellosis eradication and control programs and have been used worldwide successfully for decades. Strain RB51 and strain19 are the approved *B. abortus* vaccines most commonly used in cattle for protection against infection and abortion. However, much effort has been taken for the development of new safe and effective vaccines due to some drawbacks shown by these vaccines that used in other susceptible animals [7].

Truong *et al*. [8] used other attenuated vaccines that have been most frequently used for brucellosis; they have constructed unmarked mutants by deleting singly cydD and cydC genes, which encode adenosine 5’-triphosphate-binding cassette transporter proteins, from the virulent *B. abortus* chromosome isolate from Korean cow (referred to as IVK15), vaccination of mice with these mutant could elicit an anti-*Brucella* specific immunoglobulin G (IgG) and IgG subclass responses as well as enhance secretion of interferon-gamma (IFN-γ), and provided better protection against challenge with *B. abortus* strain 2308 than with the commercial *B. abortus* strain RB51 vaccine.

Hop *et al*. [9] used the purified recombinant organic hydroperoxide resistance (rOhr) with adjuvant by injection intraperitonially (IP) into BALB/c mice, A protective immune response revealed that rOhr induced increase significantly in both the IgG1 and IgG2a titers, and IgG2a reached a higher level than IgG1 after the second and third immunizations. In addition, immunization induced cell-mediated immunity (CMI) by production of IFN-γ as well as proinflammatory cytokines such as tumor necrosis factor (TNF), MCP-1, interleukin (IL-12p70), and IL-6, but a lesser amount of IL-10 suggesting that rOhr predominantly elicited a cell-mediated immune response. In addition, immunization with rOhr caused a significantly higher degree of protection against a virulent *B. abortus* infection compared with a positive control group consisting of mice immunized with maltose-binding protein, Dorneles *et al*. [10] reported the characteristics of the immune responses triggered by vaccination versus infection by *B. abortus*. The *B. abortus* specific immunological responses in mice, which showed an active participation of macrophages, dendritic cells, IFN-γ producing CD4 (+) T-cells, and cytotoxic CD8 (+) T-cells, are vital to overcome the infection. Dabral (2014) demonstrate the feasibility of using gamma-irradiated *Brucella* *neotomae* as an effective and a safe oral vaccine to induce protection against respiratory and systemic infections with virulent *Brucella*. These vaccines elicited serum antibodies included the isotypes of IgM, IgG1, IgG2a, IgG2b and IgG3; all oral vaccination induced antigen-specific CD4 (+) and CD8 (+) T-cells capable of secreting IFN-γ and TNF-α.

Engel *et al*. [11] reported that the immunosuppressive activity of neonicotinoids pesticides may interfere with complex microbial interactions. Dirwal *et al*. [12] study the immunotoxic effects of TMX in immunized mice with *Brucella melitensis* Rev1. The immunological examination cellular (delayed type hypersensitivity [DTH] skin test) and humoral immunity revealed that the TMX induced depressed in both arms of immune response as comparing with vaccinated non-treatment animals.

Exposure of human to IMD was occurred during its using. An investigation of the developmental immunotoxic potential of IMD was recorded. The IMD caused age-dependent adverse effects on the developing immunity, which was aggravated when continuity of exposure throughout development which leading to a compromised immune system [13].

IMD, a neonicotinoid insecticide has been in use worldwide for years in veterinary medicine. The residue of these compounds may be recycled in the food chain; evaluation of immunotoxic effects of IMD in female BALB/c mice was done by the administration of this compound orally daily at 10, 5, or 2.5 mg/kg over 28 days. The parameters of humoral and cellular immune response, including hemagglutinating antibody titer to sheep red blood cells (SRBC; T-dependent antigen), DTH response to SRBC, and T-lymphocyte proliferation in response to phytohemagglutinin (passive hemagglutination [PHA]), were evaluated. The results showed a specifically suppressed cell-mediated immune response as was evident from decreased DTH response and decreased stimulation index of T-lymphocytes to PHA. Footpad sections of mice revealed dose-related suppression of DTH response. The results indicated that IMD has immunosuppressive effects at doses >5 mg/kg, which could potentially be attributed to direct cytotoxic effects of IMD against T-cells (particularly TH cells) and that long-term exposure could be detrimental to the immune system [14].

No data are available regarding the immunotoxicity of TMX, so the present study was conducted to determine the immunotoxic effects of TMX in mice and its influence in suppression of immunity induced by *Brucella* vaccine.

## Materials and Methods

### Ethics approval

This study was approved by the Ethical and Research Committee of Veterinary Medicine College/University of Baghdad.

### Experimental animals

A total of 60 female albino mice, supplied from Animal House of the College of Veterinary Medicine, University of Baghdad. They were housed and maintained in a conventional animal facility; mice were fed on special formula of food pellets and were given water *ad libitum*. Throughout the experiment, each group of mice was housed in a plastic cage containing hardwood chip as bedding. The experiment was conducted in the University of Baghdad/College of Veterinary Medicine/Department of Pathology and Poultry.

### Determination of effective dose of TMX

TMX (25%) from Syngenta Company (Switzerland). The effective dose from lethal dose 50% (LD_50_) of TMX was measured by “up-and-down” method according to Dixon [15]; this dose was given orally to mice.

### Preparation of antigens

Brucellin antigen (soluble antigen) was prepared according to Mitov *et al*. [16]. This antigen was used in the DTH skin test. Protein concentration was measured according to biuret procedure.

Culture filtrated *B. abortus* antigen (CFBAgs) was done according to Mitov *et al*. [16]. And used for immunizing animals, this antigen was prepared by culturing of *B*. *abortus* bacteria on tryptone soya agar plate and incubated at 37°C for 7 days. Microscopic examination by Gram-stain was done to insure pure the purity of culture. Then, the culture was harvested by adding phosphate-buffered saline pH 7.2 after 10 min. The suspension was centrifuged at 30,000 rpm/4°C/30 min by cold centrifuge. The supernatant was filtered by Millipore filter 0.22 nm. Then, the filtrated fluid was examined by Gram-stain and cultured in blood agar to confirm sterility of this antigen. The total protein concentration was measured according to biuret procedure.

### Immunological tests

DTH test (skin test) was carried out at the 28^th^ day post 1^st^ immunization, and the procedure was adapted by Hudson and Hay [17].

Detection of serum IFN-γ concentration in mice sera was assessed using commercial available enzyme-linked immunosorbent assay (ELISA) Kit obtained from Cuscino Biotech Co., Ltd. (China). The results were reported as pictogram/milliliter (Pg/mL). This test was carried out according to the manufacture assay protocol.

PHA test was done according to Herbert [18]. Moreover, phagocytic assay in serum was estimated by the carbon clearance assay according to Cheng and Lamont [19] and modified by Chao and Lee [20].

### Experimental design

A total of 60 mice, aged 7-8 weeks, were divided randomly into three main groups, equally that treated as follows: The 1^st^ group (n=20) was immunized with (CFBAgs), IP in two doses at 2 weeks intervals (protein concentration 2 mg\ml). The 2^nd^ group (n=20) were immunized as in G1 group by (IP) rout and administered orally with 1/10 LD_50_ (83.7 mg\kg B.W.) of TMX daily, and the 3^rd^ group (n=20) was administrated orally with 0.3 ml of normal saline and served as a control group.

At day 28 post-immunization (PI), DTH skin test was done using brucellin soluble antigen. At 30 days PI serum samples were collected from all groups of mice and used for detection of PHA test; IFN-γ by ELISA, in addition to phagocytic assay.

### Statistical analysis

Statistical analysis was conducted to determine the statistical differences among different groups using ready-made statistical design Statistical Package for Social Science.

## Results

### DTH (skin test)

At 24 h post-test, the results showed that the mean values of skin thickness against *Brucella* were significantly (p≤0.05) high in the 1^st^ group (0.51±0.002 mm) as compared with the 2^nd^ group which showed (0.08±0.002 mm); the mean values of skin thickness were declined in the 1^st^ group (0.46±0.002) and 2^nd^ group (0.07±0.001) at 48 h. DTH skin tests indicated that the values were significantly high (p<0.05) in the immunized group compared to the control group and there is a significant effect of the antigen injected on the foot pad skin thickness of mice (Table-1).

**Table-1:** Thickness difference of footpad skin in mice after injection I/D with *B. abortus* soluble antigen.

Groups	Mean±SE	

	24 h at footpad skin thickness	48 h footpad skin thickness
1^st^	0.51±0.002^a^	0.46±0.002^a^
2^nd^	0.08±0.002^b^	0.07±0.001^b^
3^rd^	0	0

a,b Different superscripted signs in the same column indicate significant differences p≤0.05. Values are means±standard deviation. Group 1: Immunized with CFBAg, Group 2: Immunized with CFBAg and treated with TMX, Group 3: Control negative group, SE=Standard error, CFBAg=Culture filtrate *Brucella abortus* antigen, TMX=Thiamethoxam, *B. abortus*=*Brucella abortus*

### Serum IFN-γ

The current result showed that the mean values of serum IFN-γ (pg/ml), at 30-day PI in the 1^st^ mice was higher (406.36±1.52), than those values in the 2^nd^ mice (151.61±0.89) and control group (46.47±0.60), as revealed in Table-2.

**Table-2:** The mean value of serum IFN-γ-30 day PI of different mice groups immunization at 4 weeks by ELISA test.

Groups	IFN-γ (pg/ml) (mean±SE)
1^st^	406.36±1.52^a^
2^nd^	151.61±0.89^b^
3^rd^	46.47±0.60^c^

a,b,c Different superscripted signs in the same column indicate significant differences p≤0.05. Values are means±standard deviation. IFN=Interferon gamma, ELISA=Enzyme-linked immunosorbent assay, PI=Post immunization

### PHA test

The results of PHA examination at the 4 weeks PI expressed that the serum Abs titers in the G1 group was 139±12.16 significantly (p≤0.05) higher than in the G2 group (7.66±0.33), control group showed a negative results as revealed in Table-3.

**Table-3:** The antibody titers against *B. abortus* vaccine in the immunized and control group at 4 weeks by PHA in mice.

Groups	Titers of Abs at 4 weeks PI (mean±SE)	p-value
1^st^	139±12.16^a^	(p≤0.05)
2^nd^	7.66±0.33^b^	(p≥0.05)
3^rd^	0	NS

a,b Different superscripted signs in the same column indicate significant differences p≤0.05. Values are means±standard deviation. *B. abortus*=*Brucella abortus*, PHA=Passive hemagglutination, PI=Post immunization, SE=Standard error, NS=Non significant

### Phagocytes assay

The current results showed that the mean values of serum phagocyte ratio (%), 30 dayd PI in the 1^st^ group was significant (p≤0.05) higher (18.55±0.44) than the 2^nd^ group (13.24±0.32) while the negative control group represent within the normal range (5.46 ±0.25) (Table-4).

**Table-4:** Phagocyte assay ratio of different groups immunization at 30 days.

Groups	Phagocyte % after 30 days (mean±SE)
1^st^	18.55±0.44^a^
2^nd^	13.24±0.32^b^
3^rd^	5.46±0.25^c^

a,b,c Different superscripted signs in the same column indicate significant differences p≤0.05. Values are means±standard deviation. SE=Standard error

## Discussions

This study revealed that the mean values of skin thickness in immunized animals treated with TMX were lower than that value in immunized animals only. This result may indicate that CFBAgs stimulated CMI and TMX diminished the immune response elicited by this Ags. This observation also may give indication that environmental pollution by pesticides associated with decreased activity of vaccine program against brucellosis in farm animals.

The result of skin test agreed with that of serum levels of IFN-γ and supported the idea that TMX can cause immunotoxicity. Since DTH reaction was considered one arm of CMI, which was dependent on Th1 producing cytokines particular IFN-γ. This evidence is in consistence with Allen *et al*. [21] who demonstrated that CD4+ and CD8+ T-cells was a main effectors cells of CMI produced IFN-γ. This cytokines activated and attract immune cells particularly macrophages to site of Ags inoculation that lead to DTH reaction as recorded by Oliveira and Splitter [22].

On the basis of this observation, it was suggested that TMX may be inhibited proliferation and attraction of macrophages and lymphocytes to the site of examination; this idea in agreement with Ramzi *et al*. [23] who demonstrated that rats treatment with acetamiprid showed significantly decreased in total leukocytes and decrease in mean values of globulin and they suggested that this pesticide can induce suppression of both humoral and cellular immune response in the rats.

This study showed that immunized animal treated with TMX expressed significantly low levels of serum antibodies titer as compared with immunized animals only. This result may indicate that TMX induced suppression of humoral immune response, in accordance to the result of DTH reaction and serum levels of IFN-γ in this group. It was suggested that correlation between CMI and humoral immunity.

Th2 cytokines were essential factor that helps proliferation and differentiation of B-cells into plasma cells producing Abs; these mechanisms were dependent on activity of IL-2 producing by Th1 immune response [24]. Furthermore, the current suggestion is in agreement with Mohamed *et al*. [25] who reported that immunized mice with soluble *Brucella* antigens stimulated spleen cells to produce Th2 response.

In the present, CFBAgs were contain secretion products of *B. abortus* which were protein in nature that a good stimulator of CMI, this idea was agreement with Maecker *et al*. [26] who found that both CMI and humoral immune responses can elicited by surface proteins of *B. melitensis*.

According to above evidence, it was suggested that TMX alter activity of macrophage to produced IL-12 which stimulated NK-cells to produce IFN-γ, and this cytokine play crucial role in differentiation of Th0 toTh1 cells that produced IFN-γ again [27]. This investigation may be explained the reason of low-level serum of IFN-γ in immunized –TMX treatment group, in addition, it was postulated that low levels of serum antibody titers were related with low levels of serum IFN-γ. This observation was supported idea that *Brucella* OMP28 elicited Th1 producing IFN-γ stimulated production of IgG2 and Th2 response that help production IgG1 [28].

The high percentage of phagocytic activity in immunized animals as compared with those values in the control negative group may indicate that the CFBAgs were a good stimulator of phagocytic cells. This idea is in consistence with a previous study which recorded that *Brucella* antigens can stimulate macrophages to synthesize IL-12 and TNF-α resulting in stimulation of natural killer cells to synthesis of IFN-γ [10,29].

Furthermore, the current finding showed that animals treated with TMX were very significantly low in the phagocytic activity as compared with the control group. This result may indicate that TMX induced impairment of host defense mechanism through depressed activity of phagocytic cells which were considered main cells of innate immune response against infectious and non-infectious agents. This observation was an agreement with Ramzi *et al*. [23] investigated that administrated rats with 1\100 LD_50_ of IMD (IC) for 4 weeks expressed a significant decrease in phagocytic activity.

It was found in the present result that immunized animals-treatment with TMX showed low phagocytic activity as compared with immunized animals alone. This result also supported the current observation of immunotoxic effects of TMX, which induced decrease in the level of INF-γ, important stimulator of phagocytic activity or direct effects of this pesticide on bone marrow hemopoitic cells. This evidence was in consistence with Jeong *et al*. [30] who suggested that (IC) pesticides act on hematopoietic organ and prevent of macrophage arming factor uptake or increased migration inhibitor factor activity which leads to decrease mobility of phagocytic cells.

## Conclusion

This study revealed how the environmental exposure of TMX may produce suppression of humoral and cellular immune responses PI of animals with *Brucella* vaccine.

## Authors’ Contributions

MJA and AAY were involved in the design of this research work. The research was done by LHS. MJA and AAY have monitored all the activities being supervisor and revised the draft manuscript. All authors read and approved the final manuscript.

## References

[1] Maienfisch P, Angst M, Brandl F, Fischer W, Hofer D, Kayser H, Kobel W, Rindlisbacher A, Senn R, Steinemann A, Widmer H (2001). Chemistry and biology of thiamethoxam:A second generation neonicotinoid. Pest Manag. Sci.

[2] Ford K.A, Casida J.E (2008). Comparative metabolism and pharmacokinetics of seven neonicotinoid insecticides in spinach. J. Agric. Food Chem.

[3] Kurwadkar S, Evans A, DeWinne D, White P, Mitchell F (2016). Modeling photodegradation kinetics of three systemic neonicotinoids - Dinotefuran, imidacloprid andthiamethoxam in aqueous and soil environment. Environ. Toxicol. Chem.

[4] Tomizawa M, Casida J.E (2005). Neonicotinoid insecticide toxicology:Mechanisms of selective action. Annu. Rev. Pharmacol. Toxicol.

[5] Sinha S, Thaker A.M (2014). Study on the impact of lead acetate pollutant on immunotoxicity produced by thiamethoxam pesticide. Indian J. Pharmacol.

[6] Di Prisco G, Cavaliere V, Annoscia D, Varricchio P, Caprio E, Nazzi F, Gargiulo G, Pennacchio F (2013). Neonicotinoid clothianidin adversely affects insect immunity and promotes replication of a viral pathogen in honey bees. Proc. Natl. Acad. Sci. U S A.

[7] Elaine M.S, Sriranganathan D.N, Lage A.P (2015). Recent advances in *Brucella abortus* vaccines. Vet. Res.

[8] Truong Q.L, Cho Y, Park S, Park B.K, Hahn T.W (2016). *Brucella abortus* mutants lacking ATP-binding cassette transporter proteins are highly attenuated in virulence and confer protective immunity against virulent B. Abortus challenge in BALB/c mice. Microb. Pathog.

[9] Hop H.T, Reyes A.W, Simborio H.L, Arayan L.T, Min W.G, Lee H.J, Lee J.J, Chang H.H, Kim S (2016). Immunization of mice with recombinant *Brucella abortus* organic hydroperoxide resistance (Ohr) protein protects against a virulent *Brucella abortus*544 Infection. J. Microbiol. Biotechnol.

[10] Dorneles E.M, Teixeira-Carvalho A, Araújo M.S, Sriranganathan N, Lage A.P (2015). Immune response triggered by *Brucella abortus* following infection or vaccination. Vaccine.

[11] Engel P, Martinson V.G, Moran N.A (2012). Functional diversity within the simple gut microbiota of the honey bee. Proc. Natl. Acad. Sci. U S A.

[12] Dirwal A.R, Alwan M.J, Falih A.B (2014). Toxopathological and immunotoxical effects of thiamethoxam in white mice. AL-Qadisiya J. Vet. Med. Sci.

[13] Gawade L, Dadarkar S.S, Husain R, Gatne M (2013). A detailed study of developmental immunotoxicity of imidacloprid in Wistar rats. Food Chem. Toxicol.

[14] Badgujar P.C, Jain S.K, Singh A, Punia J.S, Gupta R.P, Chandratre G.A (2013). Immunotoxic effects of imidacloprid following 28 days of oral exposure in BALB/c mice. Environ. Toxicol. Pharmacol.

[15] Dixon W.J (1980). Efficient analysis of experimental observations. Annu. Res. Pharmacol. Toxicol.

[16] Mitov I, Denchen V, Linde K (1992). Humoral and cell mediated immunity in mice after immunization with live oral vaccines of *Salmonella typhimurium* anxotrophic mutants with two attenuating markers. Vaccine.

[17] Hudson L, Hay F.C (1980). Practical Immunology.

[18] Herbert W.J, Weir D.M (1978). Passive haemagglutination with special reference to the tanned cell technique. Cellular immunology. Handbook of Experimental Immunology.

[19] Cheng S, Lamont S.J (1988). Genetic analysis of immunocompetence measures in a white Leghorn chicken line. Poult. Sci.

[20] Chao C.H, Lee U.P (2001). Relationship between reproductive performance and immunity in Taiwan country chickens. Poult. Sci.

[21] Allen J.W, Shuler C.F, Mendes R.W, Latt S.A (1977). A simplified technique for *in vivo* analysis of sister chromatid exchanges using 5-bromo deoxyuridine tablets. Cytogenet. Cell Genet.

[22] Oliveira S.C, Splitter A.G (1995). CD8+Type 1 CD44hi CD45 RBlo T lymphocytes control intracellular *Brucella abortus* infection as demonstrated in major histocompatibility complex class I- And class II-deficient mice. Eur. J. Immunol.

[23] Ramzi S.C, Vinay K, Stanley R (1994). Pathologic Basis of Diseases.

[24] Khan A.L.A, Khan M.Z (2012). Hemato-biochemical changes induced by pyrethroid insecticides in avian, fish and mammalian species. Int. J. Agric. Biol.

[25] Mohamed M, Gamal B, Inas R, Mostafa E (2011). Immunological and histological effects of exposure to imidacloprid insecticide in male albino rats. Afr. J. Pharm. Pharmacol.

[26] Maecker H.T, Umetsu D.T, DeKruyff R.H, Levy S.H (1998). Cytotoxic T cell responses to DNA vaccination:Dependence on antigen presentation via class II MHC. J. Immunol.

[27] Zhan Y, Cheers C (1995). Endogenous interleukin-12 is involved in resistance to *Brucella abortus* infection. Infect. Immun.

[28] Mahajan N.K, Kulshreshtha R.C, Malik G, Dahiya J.P (2005). Immunogenicity of major cell surface protein(s) of *Brucella melitensis* Rev 1. J. Res. Commun.

[29] Hsieh C.S, Macatonia S.E, Tripp C.S, Wolf S.F, O’Garra A, Murphy K.M (1993). Development of TH1 CD4+T cells through IL-12 produced by *Listeria*-induced macrophages. Science.

[30] Jeong H.Y, Mitchell V.A, Vaughan C.W (2012). Role of 5-HT_1_ receptor subtypes in the modulation of pain and synaptic transmission in rat superficial dorsal horn. Br. J. Pharmacol.

